# Hypopigmented Mycosis Fungoides: Loss of Pigmentation Reflects Antitumor Immune Response in Young Patients

**DOI:** 10.3390/cancers12082007

**Published:** 2020-07-22

**Authors:** Amelia Martínez Villarreal, Jennifer Gantchev, François Lagacé, Augustin Barolet, Denis Sasseville, Niels Ødum, Yann Vincent Charli-Joseph, Amparo Hernández Salazar, Ivan V. Litvinov

**Affiliations:** 1 Division of Experimental Medicine, McGill University, Montreal, QC H4A3J1, Canada; amelia.martinezvillarreal@mail.mcgill.ca (A.M.V.); jennifer.theoret@mail.mcgill.ca (J.G.); 2 Division of Dermatology, McGill University, Montreal, QC H4A3J1, Canada; francois.lagace@mail.mcgill.ca (F.L.); augustin.barolet@mail.mcgill.ca (A.B.); denis.sasseville@mcgill.ca (D.S.); 3 LEO Foundation Skin Immunology Research Center, Department of Immunology and Microbiology, University of Copenhagen, DK-2200 Copenhagen, Denmark; ndum@sund.ku.dk; 4 Cutaneous Hematopathology Clinic, Dermatology Department, Instituto Nacional de Ciencias Médicas y Nutrición Salvador Zubirán, Ciudad de México 14080, Mexico; ycjderm@gmail.com (Y.V.C-J.); amton72@gmail.com (A.H.S.); 5 Department of Dermatology, University of California San Francisco, San Francisco, CA 94115, USA

**Keywords:** mycosis fungoides, cutaneous T-cell lymphomas, hypopigmentation, hypopigmented mycosis fungoides, Th1, antitumor immune response, cytotoxic cells, immunosurveillance, immunoediting

## Abstract

Hypopigmented mycosis fungoides (HMF) is a form of cutaneous T-cell lymphoma (CTCL), a heterogeneous group of extranodal non-Hodgkin’s lymphomas. HMF has a unique set of defining features that include light colored to achromic lesions, a predilection for darker skin phototypes, an early onset of disease, and predominance of CD8^+^ T-cells, among others. In the current review, we detail the known pathways of molecular pathogenesis for this lymphoma and posit that an active Th1/cytotoxic antitumor immune response in part explains why this variant is primarily seen in children/adolescents and young adults, who do not exhibit signs of immunosenescence. As a result of this potent cytotoxic response, HMF patients experience mostly favorable overall prognosis, while hypopigmentation may in fact represent a useful surrogate marker of cytotoxic immunity targeting the malignant cells. Understanding the molecular processes behind the specific features that define HMF may lead to improved diagnostic accuracy, personalized prognosis by risk stratification, and improved management of HMF. Moreover, improving our knowledge of HMF may aid our further understanding of other cutaneous lymphomas.

## 1. Introduction

Mycosis fungoides (MF) is a form of cutaneous T-cell lymphoma (CTCL), a heterogeneous group of extranodal non-Hodgkin’s lymphomas characterized by the expansion of monoclonal T-cells involving the skin [1,2,3]. MF and the leukemic disease Sézary syndrome (SS) are the two most commonly recognized forms of CTCL and account for approximately 53% of all CTCL cases [4,5]. Within MF, there are several variants, including, but not limited to, conventional Alibert-Bazin, granulomatous, granulomatous slack skin, poikilodermatous/poikiloderma vasculare atrophicans, pagetoid reticulosis, folliculotropic, syringotropic, and hypopigmented MF [6,7]. Hypopigmented mycosis fungoides (HMF) is an important variant of MF to investigate, due to its high incidence in pediatric and juvenile populations, as well as its favorable prognosis when compared to the conventional Alibert-Bazin MF [8]. 

## 2. Characteristics of Hypopigmented Mycosis Fungoides

**Figure 1:**
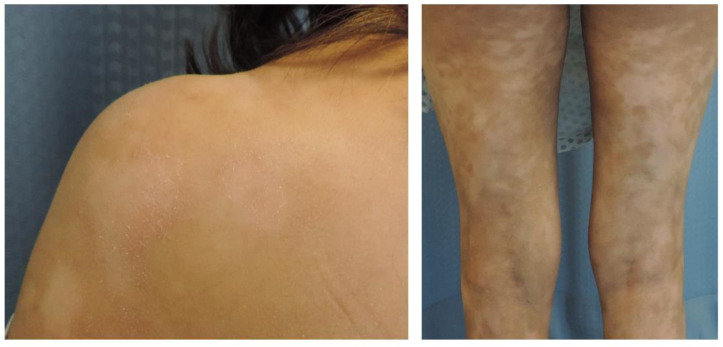
Clinical images of hypopigmented mycosis fungoides (HMF).

HMF is characterized by light colored to achromic lesions, mostly patches or thin plaques (Figure 1) [8,9]. Nonetheless, tumors have been reported in rare patients [10,11]. Currently, the most accepted hypothesis explains that the light colored to achromic skin lymphoma is due to damaged and reduced number of melanocytes in addition to abnormal melanogenesis [8,12].

HMF lesions are commonly found in non-sun exposed areas, predominantly on the extremities, the buttocks, and the trunk in a bathing-suit pattern [7,13]. There are a few documented cases of HMF with facial involvement [9]. The number, size, and shape of the lesions are variable, with some case reports ranging from a single lesion [14] to lesions covering large body surface areas, with poorly circumscribed or irregular shapes. Lesions are occasionally accompanied by pruritus, sensitivity, telangiectasia, and/or atrophy. In rare cases, systemic signs, such as lymphadenopathy, are present [8,9,15]. In this review, we classified case reports/case series of HMF cases based on whether they took the Fitzpatrick skin phototype classification into account (Supplementary Materials Table S1) or not (Supplementary Materials Table S2). In addition, we highlighted key demographic features of the disease.

HMF is more prevalent in populations with darker skin phototypes (Fitzpatrick phototypes IV–VI), including African-American, South Asian, Middle Eastern, and Hispanic individuals, where the hypopigmented skin lesions are more clinically apparent [4,9]. Nevertheless, there are reports of HMF in Caucasian populations as well [16]. There is a debate whether a female predominance exists [17]; however, most publications agree that the female to male ratio is approximately 1:1 [18]. 

HMF has an earlier age of onset than the conventional MF. Conventional Alibert-Bazin MF commonly appears in older patients with a median age between 55 and 60 years at diagnosis [7]. In contrast, the age of onset for HMF is much earlier, with several cases reported in pediatric, adolescent, and early adulthood populations. The disease has been reported in children as young as 6 months of age [19]. 

HMF has a better prognosis than the conventional MF. Stratification and clinical staging remain the best prognostic factors for conventional Alibert-Bazin MF [1], which are determined by the skin disease burden and extracutaneous involvement. Conventional MF diagnosed at an early stage (IA–IIA) often has an indolent course and slow progression [4], whereas the life expectancy for advanced stages (≥IIB) ranges between 3.2 and 9.9 years [2]. HMF, in general, has an excellent prognosis and the majority of patients are diagnosed at an early stage (IA–IB) of the disease. They present with an indolent form and they rarely progress beyond stage IB [8]. 

Like in other forms of MF, the treatment of HMF depends on the clinical stage at presentation. In most cases, a skin-directed therapy is used. Phototherapy (e.g., narrowband ultraviolet B or in select cases, ultraviolet A1) and photochemotherapy (e.g., psoralen and ultraviolet A (PUVA)) are the treatments most commonly used first-line together with topical products (e.g., topical steroids, retinoids, imiquimod, or nitrogen mustard). Photochemotherapy, also known as PUVA, involves the ingestion of 8-methoxypsoralen about 1.5–2 h prior to an exposure to ultraviolet A (UVA) radiation (320–400 nm). In younger patients with folliculotropic HMF, UVA1 (340–400 nm) treatment, if available, may be a safer alternative to PUVA. Photochemotherapy induces DNA damage, suppresses keratinocyte cytokine production, reduces the number of Langerhans cells, and induces apoptosis on malignant cells. Narrowband ultraviolet B light (NUVB; 311 nm) is the first-line method of treatment of HMF, where it works by suppressing malignant cell proliferation through increased keratinocyte cytokine production and through inhibition by antigen-presenting cells [7,8]. Often, a course of phototherapy that lasts several months to a year, together with the aforementioned topical products, enables dermatologists to achieve disease control and induce a long-lasting remission. Very rarely, in aggressive disease, a total skin electron beam therapy (TSEBT) may be considered [7]. However, since HMF patients are usually adolescents/young adults, most physicians try other treatment options before resorting to radiation. 

Regarding the topical products, topical steroids are most commonly used early in MF. Steroids modify lymphocyte adhesion to the endothelium, downregulate Nuclear factor-κB (NF-κB) and Activator protein-1 (AP-1), decrease cytokine and growth factor production, and induce apoptosis. Topical nitrogen mustard is an alkylating agent that induces DNA damage and may affect keratinocyte–Langerhans cell interactions. Retinoids and bexarotene specifically and selectively bind to retinoid receptors (Retinoid X Receptor or RXR in the case of bexarotene), which affects cell differentiation and induces apoptosis. Imiquimod activates Toll-Like Receptor-7 (TLR7) receptor signaling, leading to local interferon-α (IFN-α) and interferon-β (IFN-β) production, which augments antitumor immune response. Fortunately, HMF patients do not require systemic therapy. Some dermatologists may offer these patients a systemic retinoid (e.g., alitretinoin, isotretinoin, or bexarotene) to make the phototherapy more effective. Despite the long-term skin-directed treatments and the indolent behavior of HMF, recurrence is often reported, which may occur after months or years of total remission [8].

HMF has similar histopathological features to other MF variants. Lesions show epidermotropism of single or clusters of malignant T-cells forming Pautrier’s microabscesses, surrounding Langerhans cells. The malignant cells are haloed, small to medium in size, and have an irregular and hyperconvoluted nucleus [4]. Focal parakeratosis and spongiosis are common in HMF. A striking epidermotropism and a predominance of clonal malignant CD8^+^ T-cells are the two common histologic features of HMF, which other MF variants do not exhibit as often [6,8]. 

## 3. Immunopathogenesis of Alibert-Bazin and Hypopigmented Mycosis Fungoides

The pathogenesis of MF and HMF is incompletely understood and several theories have been proposed, highlighting the importance of external triggers, including *Staphylococcus aureus* toxins, and the activation/deregulation of JAK-STAT, NOTCH, MAPK, and other signaling pathways [3,20,21,22,23,24]. It is believed that malignant T-cells in MF arise from mature resident CD45RO^+^ T-cells engaged in normal cutaneous immune surveillance [25]. Cutaneous immune surveillance maintains homeostasis between the host and the environment. However, when this homeostasis is challenged by environmental or pathogen-driven damage to the skin, cellular injury, or stress, keratinocytes respond by releasing pro-inflammatory cytokines. These cytokines may have two consequences.

The first consequence results in the mobilization of the innate immune system. It is manifested by the recruitment of several immune cell types, such as dendritic cells (DCs), mast cells, and macrophages. These newly recruited immune cells initiate and maintain cutaneous inflammation, facilitated by the recognition of pathogen patterns, identified by their receptors such as toll-like receptors (TLRs). This recognition activates the NF-κB pathway within these immune cells, which results in direct effects on pathogens. This process constitutes the innate immune surveillance occurring in the skin [25,26].

The link between the innate and adaptive immune responses is facilitated by the activation of the NF-κB pathway in antigen-presenting cells (APCs). The specialized APCs in the epidermis are the Langerhans cells and their dermal counterparts are the dermal DCs. Research indicates that Langerhans cells may be the source of sustained antigen stimulation, resulting in a chronic inflammatory response in the skin, which is characteristic of MF. The activation of the NF-κB pathway triggers APCs to migrate toward the skin-draining lymph nodes. Once in the lymph nodes, APCs encounter naive T-cells and activate them [25]. These newly active T-cells are antigen-specific cells and express cutaneous lymphocyte antigen (CLA) as well as CC chemokine receptor 4 (CCR4), which induce a skin-targeted migration via chemotaxis. 

The second consequence of primary cytokine release by the keratinocytes directly results in the recruitment of adaptive immune cells, mostly active T-cells, in the skin. As such, the upregulation of adhesion molecules in the dermal vessels is observed. The adhesion molecules E-selectin and CC chemokine ligand 17 (CCL17) are complementary to the CLA and CCR4 receptors expressed on the newly activated T-cells, respectively. Specific recognition of these ligands allows active T-cells to tether and roll along the endothelium and extravasate into the dermis. Once in the dermis, T cells produce signaling molecules and cytokines. These signaling molecules and cytokines can have an effector function (i.e., elimination of infection) or can mediate an inflammatory response [27]. 

Once homeostasis is reestablished, the former activated T-cells should be eliminated; however, monoclonal T-cells in MF continue to proliferate [27]. Such proliferation is driven by inappropriate activation of STAT signaling pathways, upregulation of oncogenic miRNAs, activation of Thymocyte Selection-Associated HMG Box (TOX) oncogene, production of autocrine growth factors, and exposure to *S. aureus* enterotoxins [28,29,30,31,32,33,34,35,36,37,38,39,40]. Uncontrolled proliferation of active malignant T-cells (initiated by inappropriate and prolonged antigen stimulation) in the skin eventually dysregulates the normal host immune system, consequently affecting the antitumor immune response as well [25,28].

## 4. Cancer Immunoediting in Mycosis Fungoides

**Figure 2:**
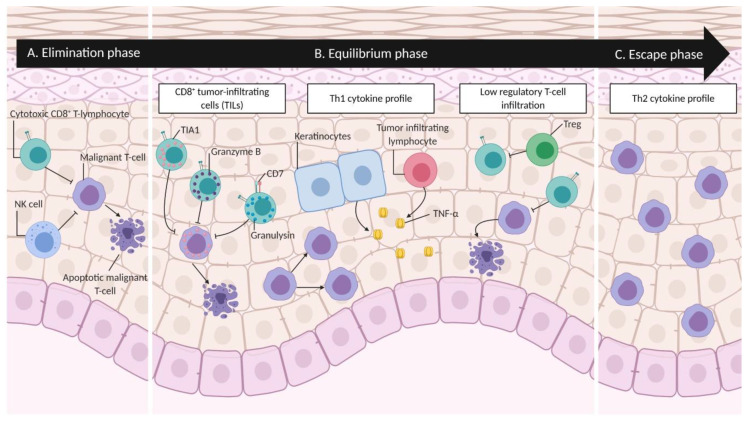
**Cancer immunoediting in mycosis fungoides.** Each phase of cancer immunoediting can be associated with specific cellular and cytokine profiles. In the elimination phase (**A**), the immune system, depicted here by cytotoxic CD8+ T-lymphocytes and Natural Killer (NK) cells, controls the proliferation of malignant T-cells, keeping them occult/incognito. Several evidences suggest that HMF remains in the equilibrium phase (**B**). Specifically, CD8+ tumor-infiltrating cells (TILs) secrete TIA1, granzyme B, and granulysin, which are cytotoxic granules that induce apoptosis in malignant T-cells. The production of Tumor Necrosis Factor-α TNF-α by keratinocytes and TILs induce an active antitumor immune response. A low level of regulatory T-cell (Treg) infiltration reported in HMF suggests that TILs are able to induce apoptosis in malignant T-cells. Finally, in the escape phase (**C**), a shift to Th2 cytokine profile allows malignant cells to overcome immune recognition and proliferate in the skin and beyond. Figure created with BioRender.com.

Uncontrolled monoclonal proliferation of active malignant T-cells homing to the skin elicits an antitumor immune response in MF [28,41]. Disease progression along with specific cellular and cytokine profiles can be associated with one of the three phases of cancer immunoediting: elimination, equilibrium, and escape (Figure 2). During the elimination phase, the immune system is able to control the proliferation of the malignant T-cells; hence, the malignant T-cells remain occult/incognito (Figure 2A) [42]. It is worth mentioning that no specific antigens or immunogenic characteristics of malignant T-cells in MF have been discovered thus far [23,24,41]. 

Progressively, the malignant cells and the immune system advance into the equilibrium phase of cancer immunoediting (Figure 2B). This phase is a period of latency characterized by the balance between surviving and dying tumor cells, sustained by the immune response [43]. Evidence of this steady state in MF includes high numbers of tumor infiltrating CD8^+^ T-cells and a Th1 cytokine profile in lesional skin. The equilibrium phase often corresponds to clinical stages IA–B and IIA in MF [41,42,44,45]. Recent research suggests that HMF remains in the equilibrium phase of the cancer immunoediting process. Research regarding cytotoxic molecules and cytokines secreted by neoplastic and/or infiltrating cells, along with the absence of infiltrating regulatory T-cells (Tregs), constitutes evidence that HMF has a better antitumor immune response, when compared to the conventional Alibert-Bazin MF. 

In conventional MF, research suggests that infiltrating CD8^+^ cytotoxic T-lymphocytes have a major role in determining disease prognosis and antitumor immune response [46]. T-cell intracytoplasmic antigen 1 (TIA1) is a cytotoxic molecule constitutively expressed by CD8^+^ cytotoxic cells, either in their active or resting state [47]. It has been reported that, in HMF, malignant CD8^+^ lymphocytes are TIA1 positive [16,17,48], whereas malignant CD8^–^ lymphocytes are TIA1 negative [49]. However, given that CD8^+^ cells express TIA1 regardless of whether they are active or resting, this does not reflect an active antitumor immune response. On the other hand, tumor-infiltrating lymphocytes (TILs), that are part of the antitumor immune response, also express TIA1 along with a series of cytotoxic cytokines (e.g., granzyme B and granulysin) [50], suggesting an active antitumor immune response.

Another cytotoxic molecule produced by active CD8^+^ cytotoxic cells is granzyme B. Granzyme B is a serine protease which induces apoptosis on its target cells [51]. The expression of granzyme B in several HMF patient samples has been demonstrated on dermal TILs, but not in malignant epidermal lymphocytes. Malignant cells do not express granzyme B, providing a possible explanation to the lack of ulceration and necrosis in HMF patients, when compared with the other forms of MF [52]. Furthermore, granzyme B positivity in dermal TILs represents a plausible evidence of the antitumor immune response that is a characteristic feature in HMF.

An analysis of granulysin, an additional cytotoxic molecule, further confirms the active involvement of antitumor immune response in HMF patients. Granulysin is expressed by activated cytotoxic lymphocytes and NK cells [53]. A differential staining between malignant cells and TILs for granulysin has been reported. Malignant CD8^+^ CD7^–^ cells often stained negatively for granulysin, while CD8^+^ CD7^+^ TILs were positive for granulysin [50]. CD8^+^ along with granulysin positivity in TILs constitutes additional support for the robust antitumor immune response provided by cytotoxic CD8^+^ tumor-infiltrating lymphocytes.

In addition to CD8^+^ TILs, a Th1 cytokine profile secreted by keratinocytes and lymphocytes provides evidence of an active antitumor immune response in HMF patients. This Th1 cytokine profile maintains the equilibrium phase of cancer immunoediting [42]. Among the cytokines that comprise the Th1 phenotype, tumor necrosis factor-α (TNF-α) has been extensively studied in HMF patients. High levels of TNF-α mRNA [54] and protein in HMF skin lesions [52] have been reported. Blocking this cytokine proves detrimental and promotes carcinogenesis and CTCL progression. Patients with hypopigmented T-cell dyscrasia, who receive TNF-α inhibitor (etanercept) treatment for rheumatoid arthritis, have been known to progress to HMF/conventional MF [55]. In summary, the same level of expression of TNF-α in hypopigmented T-cell dyscrasia and HMF suggests an active antitumor immune response. However, when this immune response is blocked, progression of carcinogenesis ensues. 

The last evidence indicating that HMF has an active antitumor immune response, which maintains the equilibrium phase of cancer immunoediting, is provided by Tregs. It has been established that Tregs inhibit natural or therapeutic immune response against tumors and can be identified by their immunophenotype CD4^+^ CD25^+^ FOXP3^+^ [43]. When compared to conventional MF, HMF presents a decreased ratio of FOXP3^+^/CD4^+^ and FOXP3^+^/CD25^+^ cells [50]. Knowing that Tregs inhibit antitumor immune responses, this decreased ratio suggests that tumor immune response is active in HMF more so than in conventional MF. How HMF patients maintain a controlled antitumor immune response has yet to be determined. 

The final phase of cancer immunoediting, the escape phase, is enabled by the genomic instability of cancer cells, activation of oncogenes, downregulation of tumor suppressor genes, ectopic reactivation of developmental/cancer testis/meiosis genes, deregulation of JAK/STAT signaling, further upregulation of TOX, contribution of external disease triggers/promoters (e.g., *S. aureus* enterotoxins), and Darwinian pressure by the immune system [28,32,56,57,58,59,60,61,62] (Figure 2C). These factors, in part, provide malignant cells with an increased resistance to immunosurveillance and promote their proliferation [43,63]. This last phase in MF is characterized by a shift to a Th2 cytokine profile with the concomitant expression of pro-eosinophilic/immunosuppressive molecules (IL-4, IL-10, and IL-13) and additional molecules such as Fas ligand, among others. This shift contributes to the escape of cancer cells from immune recognition, allowing neoplastic cells to proliferate in the skin, forming thick plaques/tumors, and beyond lymph nodes, blood, and visceral organs. [42]. 

## 5. Hypopigmentation as a Surrogate Marker of Antitumor Immune Response in MF

**Figure 3:**
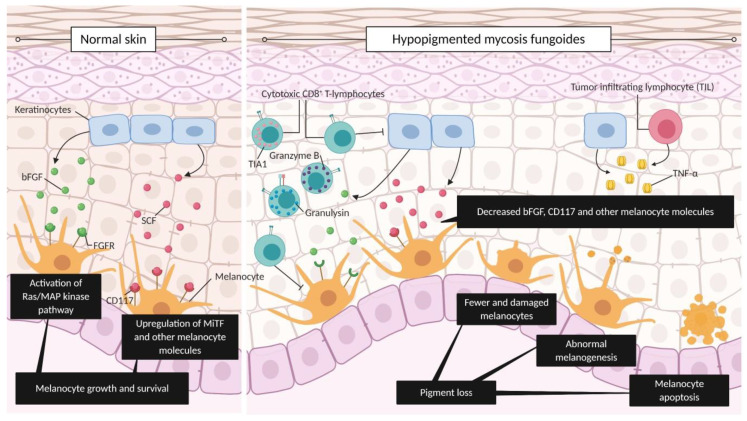
Hypopigmentation as a surrogate marker of antitumor immune response in mycosis fungoides (MF). In normal skin, keratinocytes produce basic fibroblast growth factor (bFGF) and stem cell factor (SCF). The binding of bFGF to FGF receptor (FGFR) in melanocytes activates the Ras/MAP kinase pathway, while the binding of SCF to its cognate receptor CD177 in melanocytes upregulates microphtalmia-associated transcription factor (MiTF) and additional melanocyte molecules, leading to melanogenesis. Both pathways lead to melanocyte growth and survival. In HMF, cytotoxic CD8+ lymphocytes, which act as part of the antitumor immune response, releasing granulysin and granzyme B, combined with the Th1 inflammatory response rich in TNF-α, result in damage to keratinocytes and melanocytes. This damage to keratinocytes leads to a decreased level of the melanocyte molecules bFGF and CD117, among others. Without the signals needed for melanocyte growth/survival and in the presence of granzyme B, granulysin, and TNF-α, HMF skin eventually has fewer and damaged melanocytes, abnormal melanogenesis, and apoptotic melanocytes. These features lead to the characteristic pigment loss of HMF. Figure created with BioRender.com.

Hypopigmentation in MF has been proposed to be a result of an immune response originating from neoplastic cells or reactive immune cells [12]. However, we hypothesize that hypopigmentation is caused mainly by the active antitumor immune response. Specifically, we hypothesize that the damage and alteration of melanocyte function and differentiation are results of reactive CD8^+^ cytotoxic T-lymphocytes mostly, rather than the neoplastic CD8^+^ cytotoxic T-lymphocytes. This cytotoxic activity of T lymphocytes in addition to secretion of toxic granzyme B, granulysin, and other molecules has been shown to impact two melanocyte activation pathways: the first is activated by basic fibroblast growth factor (bFGF) and the second is activated by the stem cell factor (SCF), (i.e., c-kit ligand) (Figure 3). Both factors are produced by keratinocytes and act in a paracrine fashion on melanocytes. Furthermore, a Th1 inflammatory environment rich in TNF-α likely further contributes to melanocyte damage (Figure 3).

Specifically, bFGF binds to fibroblast growth factor receptor (FGFR) expressed on melanocytes, initiating the Ras/MAP kinase signaling pathway, among others. Whereas SCF binds to the CD117 (i.e., c-kit) receptor on melanocytes, leading to the upregulation of the microphtalmia-associated transcription factor (MiTF) and other melanocyte-specific proteins. Both pathways result in melanocyte growth and survival [12,64]. When compared to normal skin and conventional MF samples, HMF has lower levels of expression of bFGF mRNA [54] and CD117, tyrosinase, MART-1/melan-A [12,65], gp100 [65], and MiTF [12] proteins. This decrease in mRNA and protein leads to pigment loss caused by fewer and damaged melanocytes, abnormal melanogenesis, and melanocyte apoptosis. The decreased levels of molecules involved in melanocyte development are independent of the predominant T-cell phenotype of the malignant cells, CD4^+^ or CD8 (Figure 3).

Furthermore, a lower level of bFGF mRNA is associated with an increased level of TNF-α in stage I HMF patients [54]. This suggests that the decreased level of bFGF, which ultimately leads to pigment loss, is associated with an active Th1 cytokine profile, as shown by increased TNF-α expression. 

Indeed, skin hypopigmentation or depigmentation is commonly associated with immune-related diseases (inflammatory dermatoses), such as Darier disease, lichen striatus, incontinentia pigmenti, pityriasis alba, and vitiligo, among others. The most accepted theory for vitiligo development is centered on an autoimmune response with CD8^+^ cytotoxic T-lymphocytes inducing melanocyte apoptosis. Specifically, infiltrating T-cells in vitiligo are associated with a Th1 phenotype, which induces melanocyte destruction via direct cytotoxicity and modulation of cytokine microenvironment [66]. Concomitant MF and vitiligo have been reported [67]; however, no studies have assessed whether vitiligo development impacts disease progression. Vitiligo patients also present a lower risk of skin cancer development, including melanoma [68]. 

Loss of pigmentation is one of the several immune-related adverse events in cancer patients under immunotherapy. These adverse events reflect an autoimmune attack on healthy tissue. Specifically, loss of pigmentation (depigmentation) has been reported in melanoma patients under immune checkpoint inhibitors and is thought to be a result of immune recognition of antigens on healthy melanocytes after breakage of immune privilege caused by malignant cell destruction [69,70]. In melanoma patients receiving either anti-programmed cell death-1 (PD-1) or cytotoxic T-lymphocyte antigen-4 (CTLA-4) therapies, the development of vitiligo-like lesions has been associated with a complete or partial response [71,72]. Moreover, tumor-infiltrating lymphocytes in these patients tend to skew towards CD8^+^ T-cell phenotype, along with increased production of interferon-γ (IFN-γ) and TNF-α [70]. Reports of vitiligo-like lesions developed after immune checkpoint therapy in patients with cancers other than melanoma are less common [73]. 

Notably, following treatment, skin re-pigmentation is a common feature in HMF patients [12]. This suggests that the depletion of neoplastic cells, along with the consequent decline in the antitumor immune response, allows the re-establishment of melanosomes and melanocytes. 

In summary, current literature suggests that hypopigmentation or depigmentation (as in vitiligo) may serve as a surrogate marker of an active immune response, specifically an antitumor immune response against malignant T-cells in MF, regardless of the T-cell phenotype. It has been established that skin cancers are highly immunogenic, reflecting the emergence of tumor-associated antigens, neoepitopes, and/or viral oncoproteins and immunosurveillance in this organ [69]. Consequently, the presence of hypopigmented patches and/or plaques in MF patients can be considered as a favorable prognostic marker.

## 6. Clinical/Demographic Patient Characteristics as a Result of Antitumor Immune Response

In addition, hypopigmentation, clinical/demographic features that are specific to HMF can be understood within the scope of active antitumor immunity found in these patients. Such clinical features include a younger age of onset and the overall good prognosis observed in HMF patients, regardless of the predominant phenotype of epidermotropic T-cells.

Childhood/juvenile MF is not common and it ranges from 2.7% to 16.6% of all MF cases. It is usually diagnosed in early stages with no lymph node involvement, and although recurrence is common, progression is unusual. Additionally, HMF is commonly overrepresented in pediatric case series [74,75,76], where patients maintain adequate immunity. 

In general, pediatric, adolescent, and early adulthood populations have a robust immune system [77], whereas elderly patients’ immune system is declining in a process termed immunosenescence. Immunosenescence is characterized by a lower number of naive T-cells in peripheral blood, reduction in diversity in the T-cell receptor (TCR) repertoire, and decreased diversity and integrity of CD4^+^ and CD8^+^ cells, among others. The innate immune response is well preserved in elderly populations; however, adaptive immune response is susceptible to deleterious changes that may enable carcinogenesis [78]. Children, adolescents, and young adults do not have a declining adaptive immune response or decreased number of CD8^+^ cells, which are the features that may have an impact on antitumor immune response.

Normally, HMF has a better prognosis than conventional MF, regardless of the predominant cell phenotype of the epidermotropic neoplastic T-cells. Patients with a T-cell phenotype different than CD8^+^ represent only 7.3% (Table 1) of all HMF cases reported (Tables S1 and S2). Almost all staged cases with an immunophenotype other than CD8^+^ present with early (≤IB) disease as well. In fact, the majority of cases that report progression of the disease still remain in the early stages (Table 1), thereby exhibiting an indolent course and slow progression [1,4]. Only two CD4^+^ patient deaths were reported, with accelerated progression [79,80]. In general, we consider that there are no critical differences regarding disease staging and prognosis between CD8^+^ HMF cases and HMF cases with other immunophenotypes. Thus, the literature indicates that the differential behavior of HMF compared to conventional MF is not caused directly by the predominant cell phenotype of the epidermotropic neoplastic T-cells. 

HMF is characterized by hypopigmented lesions; however, it can be associated with lesions of other variants of MF or with different clinical presentations (i.e., mixed MF) (Table 2). Mixed MF represents 11.6% of all reported HMF cases (Tables S1 and S2). Among all the mixed MF cases reported, three were staged as ≥II, two patients in stage IIA (early disease), and one patient in stage IVA. The latter was treated with chemotherapy and remained disease-free after 7 years of follow-up [76] (Table 2). In addition, one patient was reported to have large cell transformation (Table 2), which usually portends a worse prognosis; however, the patient responded well to phototherapy and topical steroids [81]. African-American and dark-skinned patients that presented with hypopigmented lesions had a longer overall survival rate, regardless of whether the hypopigmentation was the only clinical feature or if there were additional lesional variants of MF [82]. A previous publication concluded that mixed MF has an earlier onset than HMF, and most cases show a phenotype different than CD8^+^. Despite these differences, the authors suggested that hypopigmented lesions represent a marker of a favorable prognosis when compared with the conventional erythematous patch/plaque MF [83]. These combined results indicate that the presentation of hypopigmented lesions, solely or concomitantly with other variants of MF or other clinical presentations, represents an active antitumor immune response, and therefore portends a favorable disease prognosis. 

## 7. Conclusions

Two of the differentiating characteristics of HMF, its earlier onset and favorable prognosis, can be explained through the involvement of an active antitumor immune response. Specifically, the immunopathogenesis of MF implies an activation of the cytotoxic immune response, the adaptive antitumor immunity. Importantly, the three phases of cancer immunoediting correlate with the different cellular and cytokine profiles of MF. We suggest that HMF remains in the equilibrium phase, which is characterized by a balance between surviving and dying tumor cells. Evidence of HMF maintenance of equilibrium phase includes TILs that are positive for cytotoxic molecules such as TIA1, granzyme B, and granulysin as well as high levels of TNF-α cytokine in lesional skin, indicating a robust Th1 immune response. Additionally, lower Treg cell levels found in HMF patient skin lesions further supports the notion of active immunosurveillance. 

Furthermore, we propose that hypopigmentation in the case of HMF constitutes a surrogate clinical marker for the active antitumor immune response, and therefore a favorable prognostic indicator in these patients. Specifically, tumor-targeting immune cells are thought to cause the observed inflammatory hypopigmentation. Decreased levels of bFGF, CD117, tyrosinase, MART-1/melan-A, gp100, and MiTF indicate/lead to disrupted melanocyte function: altered melanogenesis and induction of melanocyte apoptosis. Moreover, a decreased level of bFGF is related to an increased level of the Th1 cytokine TNF-α in HMF. 

Clinically, this active immune response correlates positively with the earlier age of disease onset in HMF patients. Even though MF is not common among young individuals, HMF is overrepresented in this group, with most cases diagnosed in early stages and without progression. We speculate that there are likely many more Caucasian patients, who develop mild HMF disease and recover from it with favorable prognosis. Since the lesions are less apparent and could be confused for mild eczema (pityriasis alba), many are likely never brought to the attention of a dermatologist or biopsied to enable the diagnosis of HMF in this Fitzpatrick type I–II skin phototype patient population. 

The research presented in this review has limitations. The incidence of MF is relatively low and its exact incidence is unknown. Therefore, there is limited literature investigating this variant that may be underestimated due to misdiagnosis and clinical masquerading. The majority of publications perform immunohistochemistry, and while this approach is valid for patient samples, recent studies use a more comprehensive approach with several techniques such as genome or RNA sequencing. Finally, there is a lack of continuity regarding the research results in HMF, where biomarkers remain poorly defined/validated. Improved understanding of the molecular features that account for HMF behavior may allow dermatologists and cutaneous oncologists to accurately diagnose and prognosticate patients suffering from this disease. Furthermore, the knowledge generated by studying HMF can be applied in other MF variants, other lymphomas, or even different types of cancer. HMF is an intriguing model for the development of new targeted therapies due to the ease of accessibility to skin lesions and its overall excellent prognosis.

## Figures and Tables

**Table 1:** Cases of hypopigmented mycosis fungoides with T-cell phenotype other than CD8^+^. Cases published up to March 2020.

Study	Number of Patients	T-Cell Phenotype	Age of Onset (years)	Fitzpatrick Phototype/Color of Skin/ Ethnicity	Stage at Diagnosis	Disease Progressed
Sigal et al., 1987 [79]	1	CD4^+^	64	Caucasian	NS ^a^	Yes ^a^
Lambroza et al., 1995 [84]	1	CD4^+^	25	Trinidadian	IB	No
Moulonguet et al., 1998 [85]	1	CD4^+^	31	Caucasian/Light skinned	IA	No
Grunwald and Amichai, 1999 [86]	1	CD4^+^	12	Caucasian	NS	No
Quaglino et al., 1999 [87]	1	CD4^+^	16	White	IA	No
Qari et al., 2000 [88]	3	CD4^+^: 2 patients CD4^+^/CD8^+^: 1 patient	31 (mean)	Hispanic, Portuguese black, and African- American	NS	NS
Stone et al., 2001 [80]	1	CD4^+^	56	Black	I	Yes ^b^
Ardigo et al., 2003 [16]	5	CD4^+^: 4 CD4^+^/CD8^+^: 1	34.4 (mean)	Caucasian	NS	NS
Gulekon et al., 2005 [89]	1	CD4^+^	3	Turkish	NS	NS
Hodak et al., 2005 [90]	2	CD4^+^	1 (mean)	NS	IB and IA	1 patient (IIA)
Wain et al., 2005 [91]	2	CD56^+^	21.5 (mean)	Asian and Somalian	IA and IB	No
Onsun et al., 2006 [92]	1	CD4^+^	8	Type II	IB	No
Hodak et al., 2006 [48]	5	CD4^–^/CD8^–^	17.8 (mean at diagnosis)	NS	IA: 1 patient IB: 4 patients	No
Grover et al., 2010 [93]	1	CD4^+^	2	Indian	IB	No
Koorse et al., 2012 [94]	4	CD4^+^	NS	Indian	NS	NS
Hassab-El-Naby and El-Khalawany, 2013 [95]	9	CD4^+^	37 (mean)	Type III: 4 patients Type IV: 5 patients	IA: 7 patients IB: 2 patients	No
Zhang and Yu, 2013 [96]	1	CD4^+^/CD8^+^	9	Chinese	NS	NS
Alhumidi, 2014 [97]	4	CD4^+^: 2 patients CD4^+^/CD8^+^: 2 patients	23 (mean)	Saudi Arabian Type III	NS	No ^c^
Boulos et al., 2014 [98]	7	CD4^+^	8.8 (mean)		IA: 3 patients IB: 4 patients	1 patient (IB)
Furlan et al., 2014 [12]	4	CD4^+^	31 (mean)	Caucasian mixed race and black	IA: 1 patient IB: 2 patients IIA: 1 patient	NS
Abdel-Halin et al., 2015 [99]	8	CD4^+^: 3 patients CD4^+^/CD8^+^: 5 patients	NS	Egyptian	NS	NS
Mateeva and Kadurina, 2015 [100]	1	CD4^+^	22	Caucasian/Bulgarian descent Type III	NS	No
Rowe et al., 2016 [11]	1	CD4^+^	71	Dark skin	NS ^d^	NS
Cervini et al., 2017 [76]	8	CD4^+^: 2 patients CD4^+^/CD8^+^: 6 patients	11.8 (mean at diagnosis)	Argentinian	IA: 1 patient IB: 7 patients	Yes ^e^
Rodney et al., 2017 [9]	5	CD4^+^: 3 patients CD4^+^/CD8^+^: 2 patients	36 (mean)	African-American and African	IA: 1 patient IB: 4 patients	No
Joseph et al., 2018 [101]	1	CD4^+^	50	NS	IA	No
Vilas Boas et al., 2018 [102]	1	CD4^+^	5	Hispanic	NS	NS
Landgrave-Gomez et al., 2019 [103]	6	CD4^+^: 2 patients CD4^+^/CD8^+^: 4 patients	NS	NS	NS	NS

NS, not specified. ^a^ Staging not specified. However, since the initial diagnosis, the patient presented with lymph node involvement and after 2 years, the patient died of septicemia and bone marrow aplasia. ^b^ Disease progression to another stage not specified. After 2 years, the patient presented with erythematous plaques, nodules, and tumors and the authors suspected lymphomatous spread from MF. The patient died of acute respiratory distress syndrome. ^c^ The authors mentioned an indolent course for the cohort of patients. ^d^ Staging was not stated. However, the authors reported lymphadenopathy and tumor stage MF. ^e^ The new stage was not stated. However, the authors mentioned that two patients progressed to a higher body surface area involvement, but no systemic disease.

**Table 2:** Cases of hypopigmented mycosis fungoides concomitant with other MF variants. Cases published up to March 2020.

Study	Number of Patients	Age of Onset (years)	Fitzpatrick Phototype/Color of Skin/ Ethnicity	Other Variants of MF as Published	Stage at Diagnosis	Disease Progressed
Sigal et al., 1987 [79]	1	64	White	Erythematous papules	NS ^a^	Yes ^a^
el-Hoshy and Hashimoto, 1995 [104]	1	15	Black	Erythematous nodules	NS	No
Lambroza et al., 1995 [84]	1	21	Jamaican-American	Hyperpigmented	IB	No
Qari et al., 2000 [88]	3	25.3 (mean)	Hispanic/dark and Portuguese/black	Red papules, pink patches, erythematous, and scaly plaques	NS	No
Stone et al., 2000 [80]	1	56	Type V	Hyperpigmented macules later evolved to erythematous lesions	I	Yes ^b^
Ardigo et al., 2003 [16]	6	30.1 (mean)	Caucasian	Erythematous lesions	NS	2 patients ^c^
Ben-Amitai et al., 2003 [105]	5	4.6 (mean)	Light and pigmented	Classic erythematous lesions	IA: 2 patients IB: 3 patients	No
Wain et al., 2003 [75]	2	10.5 (mean)	NS	Poikiloderma and pilotropic	IB	No
Hodak et al., 2005 [90]	2	1 (mean)	NS	Psoriasiform	IB and IA	1 patient (IIA)
Wain et al., 2005 [91]	2	21.5 (mean)	Asian and Somalian	Poikiloderma and hyperpigmented	IA and IB	No
Hodak et al., 2006 [48]	1	12 (at diagnosis)	NS	Classic	IB	No
Hsiao et al., 2006 [106]	1	12	NS	Hyperpigmented	IA	No
Onsun et al., 2006 [92]	1	5 (mean)	Type II: 1 patient Type III: 1 patient	Erythematous	IB	No
Ozcan et al., 2008 [107]	1	30	Turkish	Erythematous	NS	NS
Cho-Vega et al., 2010 [108]	1	34	African-American	Poikiloderma	IB	No
Nanda et al., 2010 [109]	3	7.5 (mean)	Bedouin and Kuwaiti	Pityriasis lichenoides chronica-like and folliculotropic	IA, IB, and IIA	NS
Khopkar et al., 2011 [110]	5	19 (mean)	Asian with dark skin type	Poikiloderma and erythematous	NS ^d^	NS ^d^
Yazganoglu et al., 2013 [74]	8	6.1 (mean)	NS	Erythematous and purpuric	IA: 5 patients IB: 3 patients	No
Rizzo et al., 2012 [111]	1	15 (at diagnosis)	NS	Erythematous	IB	NS
Uhlenhake and Mehregan, 2012 [112]	1	49	African-American	Hypopigmented macules with hyperpigmented/erythematous centers	NS	NS
Wongpraparut and Setabutra, 2012 [113]	1	36	Type IV	Erythematous	IA	No
Ahumidi, 2014 [97]	1	5	Type III ^e^	Pink papules	NS	NS
Furlan et al., 2014 [83]	14	29.5 (median)	Mixed race, Caucasian, Black, and Asian/Brazilian	Erythematous, poikiloderma, hyperpigmented, purpuric, and hyperkeratotic	IA: 7 patients IB: 6 patients IIA: 1 patient	NS
Gameiro et al., 2014 [114]	1	5	Type III	Erythematous papules	IB	No
Heng et al., 2014 [115]	11	NS	Chinese, Malay, Indian, and others	Red, scaly papules and plaques	NS	NS
Fatemi Naeini et al., 2015 [116]	2	NS	Iranian	NS	NS	NS
Naeini et al., 2015 [117]	1	26	Iranian	Erythematous	IB	NS
Ichimura et al., 2016 [118]	1	20	Japanese	Scaly erythema	IB	No
Cervini et al., 2017 [76]	2	11 (mean at diagnosis)	Argentinian	Classic MF	IVA2^f^ and IB	No
Pradhan et al., 2017 [81]	1	2	Iranian	Large cell transformation	IB	No
Landgrave-Gomez et al., 2019 [103]	NS	NS	Hispanic	Hyperpigmentation and erythema	NS	NS
Valencia Ocampo et al., 2019 [119]	5	7.8 (mean)	Type II: 1 patient Type III: 1 patient Type IV: 2 patients Type V: 1 patient	Erythematous	IA: 1 patient IB: 4 patients	No
Geller et al., 2019 [82]	34	NS	African-American	Erythematous and hyperpigmented	NS	No
Kalay et al., 2020 [120]	4	30.5 (mean at diagnosis)	Turkish	Follicular hyperkeratosis, erythematous, and hyperpigmented	IA	No

NS, not specified. ^a^ Staging not specified. However, since the initial diagnosis, the patient presented with lymph node involvement and after 2 years, the patient died of septicemia and bone marrow aplasia. ^b^ Disease progression to another stage not specified. After 2 years, the patient presented with erythematous plaques, nodules, and tumors and the authors suspected lymphomatous spread from MF. The patient died of acute respiratory distress syndrome. ^c^ Progressive disease, but no new stage mentioned. ^d^ The authors mentioned that patients did not show lymph enlargement or visceral involvement and remained with same clinical characteristics. ^e^ The authors did not report phototype for each patient. However, the authors mentioned that Saudi Arabian individuals are mostly skin phototype III–IV. ^f^ Lymph node involvement documented. However, this patient had a follow up of 7.5 years and remained disease-free for 4 years without progression.

## Supplementary Materials

The following are available online at https://www.mdpi.com/2072-6694/12/8/2007/s1, Table S1: Hypopigmented mycosis fungoides with Fitzpatrick phototype classification. Cases published up to March 2020. Table S2: Hypopigmented mycosis fungoides without Fitzpatrick phototype classification. Cases published up to March 2020.
