# Supplementary material for: Hypopigmented Mycosis Fungoides: Loss of Pigmentation Reflects Antitumor Immune Response in Young Patients
Source: Cancers (Basel). 2020 Jul 22;12(8):2007. doi: 10.3390/cancers12082007 (PMC7465783; doi:10.3390/cancers12082007)
Supplement: Supplementary file 1 [file cancers-12-02007-s001.pdf]

Supplementary Materials: Hypopigmented Mycosis Fungoides: Loss of Pigmentation Reflects Antitumor Immune Response in Young Patients

Amelia Martínez Villarreal, Jennifer Gantchev, François Lagacé, Augustin Barolet, Denis Sasseville, Niels Ødum, Yann Vincent Charli-Joseph, Amparo Hernández Salazar and Ivan V. Litvinov

Tables S1 and S2 present case reports and articles that were available through PubMed/Medline and Web of Science searches for "All Fields∣ under the term "hypopigmented mycosis fungoides". All fields include title, journal, terms, and abstract, among others. The authors of these publications reported cases of hypopigmented lesions diagnosed as hypopigmented mycosis fungoides (HMF), whether they presented solely as hypopigmentation or concomitant with other mycosis fungoides (MF) variants. Up to March 2020, approximately 1075 HMF cases have been published. Publications with cases of HMF have been sorted into studies that report the Fitzpatrick skin phototype (250 patients, Table S1) and those that do not report the Fitzpatrick skin phototype (825 patients, Table S2). Key demographic features relevant to the disease are reported as well. In case both age of onset and diagnosis were published, age of onset was chosen. Country of origin and skin color are reported as initially published.

Table S1. Hypopigmented Mycosis Fungoides with Fitzpatrick phototype classification. Cases published up to March 2020.

Study	Number of Cases	Country of Origin/Ethnicity	Fitzpatrick Phototype	Age of Onset (Years)
Ratnam and Pang, 1994 [1]	10	Chinese, Indian and Malayian	Type II: 2<br>Type III: 4<br>Type IV: 4	19 (mean)
Stone et al, 2001 [2]	7	NS	Type IV and V	35 (mean)
Gathers et al, 2002 [3]	7	NS	Type I-III: 12<br>Type IV-VI: 12	39.5 (mean)
Al-Ratrout et al, 2006 [4]	1	Saudi Arabian	Type IV	18
Onsun et al, 2006 [5][a]	2	Turkish	Type II: 1<br>Type III: 1	5 (mean)
Pope et al, 2010 [6][a]	13	Canadian, United States/American and Australian	Type I-III: 3<br>Type IV-VI: 10	5.8 (mean)
Ranawaka et al, 2001 [7]	5	NS	Type V: 5	22.4 (mean)
AlGhamdi et al, 2012 [8]	18	Saudi Arabian	Types IV and V	25 (mean at diagnosis)
Kanokrungsee et al, 2012 [9]	11	Thailand residents	Type IV: 9<br>Type V: 2	37 (mean)
Wongpraparut and Setabutra, 2012 [10]	9	Thailand residents	Type III: 1<br>Type IV: 6<br>Type V: 2	38.2 (mean)
Hassab-El-Naby and El-Khalawany, 2013 [11]	27	Egyptian	Type II: 1<br>Type III: 9<br>Type IV: 17	35.39 (mean)
Alhumidi, 2014 [12]	17	Saudi Arabian	Type III [b]	17 (mean)
Boulos et al, 2014 [13][a]	17	Caucasian, Hispanic, African American and Middle Eastern	Types III or greater: 73%[c]	8.5 (mean)
Gameiro et al, 2014 [a] [14]	1	Caucasian	Type III	5
Hodak et al, 2014 [b] [15]	29	Israeli	Type II to III: 43%<br>Type IV to V: 88.9% [c]	6.2 (mean)
Laws et al, 2014 [16][b]	22	NS	Type II: 12%<br>Type III: 19%<br>Type IV: 27%<br>Type V: 42% [c]	11.6 (mean at diagnosis) [c]
Mateeva and Kadurina, 2015 [17]	1	Caucasian/Bulgarian descent	Type III	26
Pena-Romero et al, 2016 [18]	24	Hispanic	Type III-IV	24.6 (mean)
Tern and Gass, 2018 [19]	1	Afro-Caribbean	Type VI	6
Landgrave-Gomez et al, 2019 [20]	16	Hispanic	Type III: 3<br>Type IV: 13	NS
Valencia Ocampo et al, 2019 [21] [a]	12	NS	Type II: 3<br>Type III: 4<br>Type IV: 4<br>Type V: 1	7.9 (mean)

NS, Not specified. [a] Publication in which authors make emphasis on pediatric, adolescent and early adulthood presentation. [b] The authors do not report phototype for each patient. However it is mentioned that Saudi Arabia

residents are mostly skin phototype III–IV. [c] This data is reported for the whole cohort, without distinction between hypopigmented and other MF variants.

Table S2. Hypopigmented Mycosis Fungoides without Fitzpatrick phototype classification. Cases published up to March 2020.

Study	Number of Cases	Country of Origin/Ethnicity	Age of Onset (Years)
Ryan et al, 1973 [22]	1	NS	NS
Smith and Samman, 1978 [23]	1	West Indian	4
Breathnach et al, 1982 [24]	4 [c]	Mauritian, dark skinned Asian and Jamaican	4.7 (mean at diagnosis)
Zackheim et al, 1982 [25]	3	Black and Latin American	38 (mean)
Rustin et al, 1986 [26]	2	Venezuelian and Indian	5.5 (mean)
Goldberg et al, 1986 [27]	1	Puerto Rico/medium-brown	22
Misch et al, 1987 [28]	1	West Indian	12
Sigal et al, 1987 [29]	1	Caucasian	64 (at diagnosis)
Handfield-Jones et al, 1992 [30]	1	Jamaican	11
Volkenandt et al, 1993 [31]	1	Brown skin [a]	32
Whitmore et al, 1994 [32]	3	African-American	15.3 (mean at diagnosis)
el-Hoshy and Hashimoto, 1995 [33] [b]	1	Black skin	15
Lambroza et al, 1995 [34]	7	Jamaican-American, Trinidadian, Liberian, Puerto Rican and African-American	28.7 (mean)
Amichai et al, 1996 [35]	1	Caucasian	27
Di Landro et al, 1997 [36] [b]	1	Caucasian	7
Zakcheim et al, 1997 [37] [b]	4 [c]	Black, East Indian and Filipino with dark-brown skin and medium-complected white	9.6 (mean)
Moulonguet et al, 1998 [38]	1	Caucasian/Light-skin	31
Grunwald and Amichai, 1999 [39] [b]	1	Caucasian	12 (at diagnosis)
Quaglino et al, 1999 [40] [b]	1	White	16
Zucker-Franklin at el, 1999 [41] [b]	1	Grenada/Black skin	4
Akaraphanth et al, 2000 [42]	9	African-American and Thai	25.2 (mean)
Choe et al, 2000 [43]	1	NS	19
Hodak et al, 2000 [44]	1	Caucasian	7
Neuhaus et al, 2000 [45] [b]	1	Black skin	10 (mean)

Table S2. *Cont.*

Qari et al, 2000 [46]	5	Hispanic/dark, Portuguese/black and African-American	27.2 (mean)
Tan et al, 2000 [47] [b]	8	Chinese, Indian and Malay	9.12 (mean)
Whittam et al, 2000 [48] [b]	1	Black skin	18
El-Shabrawi-Caelen et al, 2002 [49]	15	Asian, East Indian, African-American, White, Hispanic, Native-American Indian and Ethiopian	20.6 (mean)
Ardigo et al, 2003 [50]	6 [c]	Caucasian	30.16 (mean)
Ben-Amitai et al, 2003 [51] [b]	7	Pigmented and Light skin	5.6 (mean)
Capizzi et al, 2003 [52] [b]	1	Italy/Caucasian dark skin	11
Wain et al, 2003 [53] [b]	8	Caucasian, Asian, African-Caribbean	8.25 (mean)
Das and Gangopadhyay, 2004 [54]	1	Indian	16
Fink-Puches et al, 2004 [55] [b]	2	NS	NS
Gulekon et al, 2005 [56][b]	1	Turkish	3
Hodak et al, 2005 [57]	2	NS	1 (mean)
Roupe, 2005 [58] [b]	1	Sweden/White skin	5
Wain et al, 2005 [59]	2	Asian and Somalian	21.5 (mean)
Hodak et al, 2006 [60]	5	NS	17.8 (mean at diagnosis)
Hsiao et al, 2006 [61]	1	NS	12
Manzur and Zaidi, 2006 [62] [b]	1	Pakistanian	10
Tan et al, 2006 [63]	47	Chinese, Malay, Indian	21.6 (mean)
Rodriguez et al, 2007 [64] [b]	1	Colombian	11
Chuang et al, 2008 [65]	1	African-American	41
Ozcan et al, 2008 [66]	1	Turkish	30
Kim et al, 2009 [67] [b]	5	NS	12.2 (mean at diagnosis)
Ngo et al, 2009 [68] [b]	1	Hispanic	6 months
Alsaleh et al, 2010 [69] [d]	77	Kuwaiti, Bedouin, Iraqi, Lebanese, Saudi and Egyptian	27.6 (mean at diagnosis)
Cho-Vega et al, 2010 [70]	2	African-American	13.5 (mean)
Costa and Queiroz Zancanaro, 2010 [71]	1	Caucasian	55
Lawrence et al, 2010 [72] [b]	1	African-American	14

Table S2. *Cont.*

Nanda et al, 2010 [73] [b, d]	24	Kuwaiti, Bedouin, Iraqi, Lebanese, Saudi and Egyptian	8.6 (mean at diagnosis)
Grover et al, 2010 [74]	1	Indian	2
Khopkar et al, 2011 [75]	15	Asian with dark skin type	32.2 (mean at diagnosis)
Yazganoglu et al, 2013 [76] [b]	9	NS	6.2 (mean)
Rueda et al, 2011 [77]	13	NS	NS
Koorse et al, 2012 [78]	15	Indian	NS
Rizzo et al, 2012 [79] [b]	5	Brown, white and black	10.6 (mean at diagnosis)
Uhlenhake and Mehregan, 2012 [80]	1	African-American	49
Castano et al, 2013 [81] [b]	50	African-American, Hispanic, Caucasian and Asian	NS
Juhas and English, 2013 [82]	1	NS	54
Seif El Nasr et al, 2013 [83]	14	NS	21.3 (mean at time of study)
Zhang and Yu, 2013 [84]	1	Chinese	20
Furlan et al, 2014 [85] [d]	18	Mixed race, Caucasian, Asian and Black	29.5 (mean)
Furlan et al, 2014 [86] [d]	34	Mixed race, Caucasian, Black and Asian/Brasilian	27 (mean)
Heng et al, 2014 [87] [b]	42	Chinese, Malay, Indian and others	NS
Jimenez Gallo et al, 2014 [88]	1	NS	35 (at diagnosis)
Fatemi et al, 2015 [89]	5	Iranian	25.6 (mean at diagnosis)
Abdel-Halim et al, 2015 [90]	16	Egyptian	21.31 (mean at diagnosis)
Naeini et al, 2015 [91]	1	Iranian	26
Amin et al, 2016 [92]	1	African-American	31
Ichimura et al, 2016 [93]	2	Japanese	17 (mean)
Khader et al, 2016 [94]	2	Indian	14.5 (mean at diagnosis)
Nasu-Tababuchi et al, 2016 [95]	1	Japanese	11
Paton et al, 2016 [96]	1	NS	16
Patraquim et al, 2016 [97] [b]	1	Caucasian	4
Rowe et al, 2016 [98]	1	Dark skin [a]	71
Bisherwal et al, 2017 [99]	1	Indian	25
Cervini et al, 2017 [100] [b]	14	Argentinian	11.23 (mean at diagnosis)
Pradhan et al, 2017 [101] [b]	1	Iranian	2
Rodney et al, 2017 [102]	20	African-American, African, Hispanic and Afro-Caribbean	32.2 (mean)
Virmani et al, 2017 [103][b]	27	African-American, Asian, Hispanic and Caucasian	NS
Binamer 2017 [104]	19	Saudi Arabian	NS
Martinez-Escala et al, 2017 [105]	13	African-American	NS

Table S2. *Cont.*

Abdolkarimi et al, 2018 [106][b]	1	NS	10 (at diagnosis)
Amorim et al 2018 [107]	20	White, Mixed and Black	43.85 (mean at diagnosis)
Ferreira et al, 2019 [108]	1	White	12
Stierman and Bedford-Lyon, 2018 [109]	1	NS	39
Joseph et al, 2018 [110]	1	NS	43
Vilas Boas et al, 2018 [111][b]	1	Hispanic	5
Yang et al, 2018 [112]	1	Korean	10 (at diagnosis)
Youssef et al, 2018 [113]	9	NS	17.78 (mean at diagnosis)
Park et al, 2018 [114]	1	Korean	21
Ito et al, 2019 [115]	1	NS	55
Jaque et al, 2019 [116]	10	NS	NS
Lim et al, 2019 [117]	78	Chinese, Indian, Malay and Caucasian	36.4 (mean at diagnosis)
Chen et al, 2019 [118]	1	Chinese	2
Geller et al, 2019 [119]	71	African-American, Black and Hispanics	NS
Kalay Yildizhan et al, 2020 [120]	5	Turkish	28 (mean at diagnosis)

NS, not specified, [a] Skin color of patient not reported. However, pictures in the figures show the color of the skin. [b] Publication in which authors make emphasis on childhood, pediatric, adolescent and early adulthood presentations of MF. [c] Data on one patient was published before. [d] These case reports are from the same Health Centre of patients seen and treated in the same time period. However, it is not clear whether the publications have overlap of patients.

References:

1. Ratnam, K.V.; Pang, B.K. Clinico-pathological study and five-year follow-up of 10 cases of hypopigmented mycosis fungoides. *J. Eur. Acad. Dermatol. Venereol.* **1994**, *3*, 505-510, doi:10.1111/j.1468-3083.1994.tb00409.x.
2. Stone, M.L.; Styles, A.R.; Cockerell, C.J.; Pandya, A.G. Hypopigmented mycosis fungoides: a report of 7 cases and review of the literature. *Cutis* **2001**, *67*, 133-138.
3. Gathers, R.C.; Scherschun, L.; Malick, F.; Fivenson, D.P.; Lim, H.W. Narrowband UVB phototherapy for early-stage mycosis fungoides. *J. Am. Acad. Dermatol.* **2002**, *47*, 191-197, doi:10.1067/mjd.2002.120911.
4. Al-Ratrout, J.; Al-Nazer, M.; Ansari, N. Hypopigmented mycosis fungoides in a twenty-year-old Saudi woman with fair skin. **2006**, *51*, 115-117, doi:10.4103/0019-5154.26932.
5. Onsun, N.; Kural, Y.; Su, O.; Demirkesen, C.; Buyukbabani, N. Hypopigmented mycosis fungoides associated with atopy in two children. *Pediatr. Dermatol.* **2006**, *23*, 493-496, doi:10.1111/j.1525-1470.2006.00291.x.
6. Pope, E.; Weitzman, S.; Ngan, B.; Walsh, S.; Morel, K.; Williams, J.; Stein, S.; Garzon, M.; Knobler, E.; Lieber, C., et al. Mycosis fungoides in the pediatric population: report from an international Childhood Registry of Cutaneous Lymphoma. *J. Cutan. Med. Surg.* **2010**, *14*, 1-6, doi:10.2310/7750.2009.08091.
7. Ranawaka, R.R.; Abeygunasekara, P.H.; de Silva, M.V. Hypopigmented mycosis fungoides in type v skin: a report of 5 cases. *Case Rep. Dermatol. Med.* **2011**, *2011*, 190572, doi:10.1155/2011/190572.
8. AlGhamdi, K.M.; Arafah, M.M.; Al-Mubarak, L.A.; Khachemoune, A.; Al-Saif, F.M. Profile of mycosis fungoides in 43 Saudi patients. *Ann. Saudi Med.* **2012**, *32*, 283-287, doi:10.5144/0256-4947.2012.283.
9. Kanokrungsee, S.; Rajatanavin, N.; Rutnin, S.; Vachiramon, V. Efficacy of narrowband ultraviolet B twice weekly for hypopigmented mycosis fungoides in Asians. *Clin. Exp. Dermatol.* **2012**, *37*, 149-152, doi:10.1111/j.1365-2230.2011.04197.x.
10. Wongpraparut, C.; Setabutra, P. Phototherapy for hypopigmented mycosis fungoides in Asians. *Photodermatol. Photoimmunol. Photomed.* **2012**, *28*, 181-186, doi:10.1111/j.1600-0781.2012.00662.x.
11. Hassab-El-Naby, H.M.; El-Khalawany, M.A. Hypopigmented mycosis fungoides in Egyptian patients. *J. Cutan. Pathol.* **2013**, *40*, 397-404, doi:10.1111/cup.12093.
12. Alhumidi, A.A. Hypopigmented mycosis fungoides in Saudi Arabia, epidemiological and pathological study. *J. Dermatol. Dermatol. Surg.* **2014**, *18*, 8-12, doi:https://doi.org/10.1016/j.jssdds.2013.12.002.
13. Boulos, S.; Vaid, R.; Aladily, T.N.; Ivan, D.S.; Talpur, R.; Duvic, M. Clinical presentation, immunopathology, and treatment of juvenile-onset mycosis fungoides: a case series of 34 patients. *J. Am. Acad. Dermatol.* **2014**, *71*, 1117-1126, doi:10.1016/j.jaad.2014.07.049.
14. Gameiro, A.; Gouveia, M.; Tellechea, O.; Moreno, A. Childhood hypopigmented mycosis fungoides: a commonly delayed diagnosis. *BMJ Case Rep.* **2014**, *2014*, doi:10.1136/bcr-2014-208306.
15. Hodak, E.; Amitay-Laish, I.; Feinmesser, M.; Davidovici, B.; David, M.; Zvulunov, A.; Pavlotsky, F.; Yaniv, I.; Avrahami, G.; Ben-Amitai, D. Juvenile mycosis fungoides: cutaneous T-cell lymphoma with frequent follicular involvement. *J. Am. Acad. Dermatol.* **2014**, *70*, 993-1001, doi:10.1016/j.jaad.2013.12.029.
16. Laws, P.M.; Shear, N.H.; Pope, E. Childhood mycosis fungoides: experience of 28 patients and response to phototherapy. *Pediatr. Dermatol.* **2014**, *31*, 459-464, doi:10.1111/pde.12338.
17. Mateeva, V.; Kadurina, M. Clinical, Histological and Immunohistochemical Changes in Hypopigmented Mycosis Fungoides in Response to Narrow-Band UVB Phototherapy. *J. Pigment. Disord.* **2015**, *2*, doi:10.4172/2376-0427.1000167.
18. Pena-Romero, A.G.; Montes de Oca, G.; Fierro-Arias, L.; Arellano-Mendoza, I.; Peniche-Castellanos, A.; Mercadillo-Perez, P. Micosis fungoide hipopigmentada: Diferencias clínico-histopatológicas con respecto a la micosis fungoide en placas. *Dermatol. Rev. Mex.* **2016**, *60*, 387-396.
19. Tern, P.J.W.; Gass, J. Case report: photo-onycholysis after PUVA treatment for hypopigmented mycosis fungoides with response to topical steroid. *Clin. Case Rep.* **2018**, *6*, 267-268, doi:10.1002/ccr3.1289.
20. Landgrave-Gomez, I.; Ruiz-Arriaga, L.F.; Toussaint-Caire, S.; Vega-Memije, M.E.; Lacy-Niebla, R.M. Epidemiological, clinical, histological, and immunohistochemical study on hypopigmented epitheliotropic T-cell dyscrasia and hypopigmented mycosis fungoides. *Int. J. Dermatol.* **2019**, 10.1111/ijd.14501, doi:10.1111/ijd.14501.
21. Valencia Ocampo, O.J.; Julio, L.; Zapata, V.; Correa, L.A.; Vasco, C.; Correa, S.; Velasquez-Lopera, M.M. Mycosis Fungoides in Children and Adolescents: A Series of 23 Cases. *Actas Dermosifiliogr.* **2019**, 10.1016/j.ad.2019.04.004, doi:10.1016/j.ad.2019.04.004.

22. Ryan, E.A.; Sanderson, K.V.; Bartak, P.; Samman, P.D. Can mycosis fungoides begin in the epidermis? A hypothesis. *Br. J. Dermatol.* **1973**, *88*, 419-429.
23. Smith, N.P.; Samman, P.D. Mycosis fungoides presenting with areas of cutaneous hypopigmentation. *Clin. Exp. Dermatol.* **1978**, *3*, 213-216, doi:10.1111/j.1365-2230.1978.tb01489.x.
24. Breathnach, S.M.; McKee, P.H.; Smith, N.P. Hypopigmented mycosis fungoides: report of five cases with ultrastructural observations. *Br. J. Dermatol.* **1982**, *106*, 643-649.
25. Zackheim, H.S.; Epstein, E.H., Jr.; Grekin, D.A.; McNutt, N.S. Mycosis fungoides presenting as areas of hypopigmentation: a report of three cases. *J. Am. Acad. Dermatol.* **1982**, *6*, 340-345, doi:10.1016/s0190-9622(82)70026-x.
26. Rustin, M.H.A.; Griffiths, M.; Ridley, C.M. The immunopathology of hypopigmented mycosis fungoides*. *Clin. Exp. Dermatol.* **1986**, *11*, 332-339, doi:10.1111/j.1365-2230.1986.tb00472.x.
27. Goldberg, D.J.; Schinella, R.S.; Kechijian, P. Hypopigmented mycosis fungoides. Speculations about the mechanism of hypopigmentation. *Am. J. Dermatopathol.* **1986**, *8*, 326-330.
28. Misch, K.J.; Maclennan, K.A.; Marsden, R.A. Hypopigmented mycosis fungoides. *Clin. Exp. Dermatol* **1987**, *12*, 53-55, doi:10.1111/j.1365-2230.1987.tb01858.x.
29. Sigal, M.; Grossin, M.; Laroche, L.; Basset, F.; Aitken, G.; Haziza, J.L.; Belaich, S. Hypopigmented mycosis fungoides. *Clin. Exp. Dermatol.* **1987**, *12*, 453-454, doi:10.1111/j.1365-2230.1987.tb01948.x.
30. Handfield-Jones, S.E.; Smith, N.P.; Breathnach, S.M. Hypopigmented mycosis fungoides. *Clin. Exp. Dermatol.* **1992**, *17*, 374-375, doi:10.1111/j.1365-2230.1992.tb00238.x.
31. Volkenandt, M.; Soyer, H.P.; Cerroni, L.; Koch, O.M.; Atzpodien, J.; Kerl, H. Molecular detection of clone-specific DNA in hypopigmented lesions of a patient with early evolving mycosis fungoides. *Br. J. Dermatol.* **1993**, *128*, 423-428.
32. Whitmore, S.E.; Simmons-O'Brien, E.; Rotter, F.S. Hypopigmented mycosis fungoides. *Arch. Dermatol.* **1994**, *130*, 476-480.
33. el-Hoshy, K.; Hashimoto, K. Adolescence mycosis fungoides: an unusual presentation with hypopigmentation. *J. Dermatol.* **1995**, *22*, 424-427, doi:10.1111/j.1346-8138.1995.tb03417.x.
34. Lambroza, E.; Cohen, S.R.; Phelps, R.; Lebwohl, M.; Braverman, I.M.; DiCostanzo, D. Hypopigmented variant of mycosis fungoides: demography, histopathology, and treatment of seven cases. *J. Am. Acad. Dermatol.* **1995**, *32*, 987-993, doi:10.1016/0190-9622(95)91337-8.
35. Amichai, B.; Grunwald, M.H.; Avinoach, I.; Halevy, S. Hypopigmented mycosis fungoides in a white female. *J. Dermatol.* **1996**, *23*, 425-426, doi:10.1111/j.1346-8138.1996.tb04046.x.
36. Di Landro, A.; Marchesi, L.; Naldi, L.; Motta, T.; Cainelli, T. A case of hypopigmented mycosis fungoides in a young Caucasian boy. *Pediatr. Dermatol.* **1997**, *14*, 449-452, doi:10.1111/j.1525-1470.1997.tb00687.x.
37. Zackheim, H.S.; McCalmont, T.H.; Deanovic, F.W.; Odom, R.B. Mycosis fungoides with onset before 20 years of age. *J. Am. Acad. Dermatol.* **1997**, *36*, 557-562, doi:10.1016/s0190-9622(97)70243-3.
38. Moulonguet, I.; Robert, C.; Baudot, N.; Flageul, B.; Dubertret, L. Hypopigmented mycosis fungoides in a light-skinned woman. *Br. J. Dermatol.* **1998**, *139*, 341-343, doi:10.1046/j.1365-2133.1998.02379.x.
39. Grunwald, M.H.; Amichai, B. Localized hypopigmented mycosis fungoides in a 12-year-old caucasian boy. *J. Dermatol.* **1999**, *26*, 70-71, doi:10.1111/j.1346-8138.1999.tb03513.x.
40. Quaglino, P.; Zaccagna, A.; Verrone, A.; Dardano, F.; Bernengo, M.G. Mycosis fungoides in patients under 20 years of age: report of 7 cases, review of the literature and study of the clinical course. *Dermatology* **1999**, *199*, 8-14, doi:10.1159/000018196.
41. Zucker-Franklin, D.; Kosann, M.K.; Pancake, B.A.; Ramsay, D.L.; Soter, N.A. Hypopigmented mycosis fungoides associated with human T cell lymphotropic virus type I tax in a pediatric patient. *Pediatrics* **1999**, *103*, 1039-1045, doi:DOI 10.1542/peds.103.5.1039.
42. Akaraphanth, R.; Douglass, M.C.; Lim, H.W. Hypopigmented mycosis fungoides: treatment and a 6(1/2)-year follow-up of 9 patients. *J. Am. Acad. Dermatol.* **2000**, *42*, 33-39, doi:10.1016/s0190-9622(00)90006-9.
43. Choe, Y.B.; Park, K.C.; Cho, K.H. A case of hypopigmented mycosis fungoides. *J. Dermatol.* **2000**, *27*, 543-546, doi:10.1111/j.1346-8138.2000.tb02224.x.
44. Hodak, E.; Phenig, E.; Amichai, B.; Feinmesser, M.; Kuten, A.; Maron, L.; Sahar, D.; Bergman, R.; David, M. Unilesional mycosis fungoides: a study of seven cases. *Dermatol.* **2000**, *201*, 300-306, doi:10.1159/000051542.
45. Neuhaus, I.M.; Ramos-Caro, F.A.; Hassanein, A.M. Hypopigmented mycosis fungoides in childhood and adolescence. *Pediatr. Dermatol.* **2000**, *17*, 403-406.

46. Qari, M.S.; Li, N.; Demierre, M.F. Hypopigmented mycosis fungoides: case reports and literature review. *J. Cutan. Med. Surg.* **2000**, *4*, 142-148, doi:10.1177/120347540000400306.
47. Tan, E.; Tay, Y.K.; Giam, Y.C. Profile and outcome of childhood mycosis fungoides in Singapore. *Pediatr Dermatol.* **2000**, *17*, 352-356, doi:10.1046/j.1525-1470.2000.017005352.x.
48. Whittam, L.R.; Calonje, E.; Orchard, G.; Fraser-Andrews, E.A.; Woolford, A.; Russell-Jones, R. CD8-positive juvenile onset mycosis fungoides: an immunohistochemical and genotypic analysis of six cases. *Br. J. Dermatol.* **2000**, *143*, 1199-1204.
49. El-Shabrawi-Caelen, L.; Cerroni, L.; Medeiros, L.J.; McCalmont, T.H. Hypopigmented mycosis fungoides: frequent expression of a CD8+ T-cell phenotype. *Am. J. Surg. Pathol.* **2002**, *26*, 450-457.
50. Ardigo, M.; Borroni, G.; Muscardin, L.; Kerl, H.; Cerroni, L. Hypopigmented mycosis fungoides in Caucasian patients: a clinicopathologic study of 7 cases. *J. Am. Acad. Dermatol.* **2003**, *49*, 264-270.
51. Ben-Amitai, D.; Michael, D.; Feinmesser, M.; Hodak, E. Juvenile mycosis fungoides diagnosed before 18 years of age. *Acta Dermato-venereol.* **2003**, *83*, 451-456, doi:10.1080/00015550310020530.
52. Capizzi, R.; Rotoli, M.; Cavalieri, S.; Amerio, P. Hypopigmented mycosis fungoides in a 12-year-old caucasian girl with solely hypopigmented lesions. *Dermatology* **2003**, *207*, 201-202, doi:10.1159/000071797.
53. Wain, E.M.; Orchard, G.E.; Whittaker, S.J.; Spittle, M.S.M.F.; Russell-Jones, R. Outcome in 34 patients with juvenile-onset mycosis fungoides: a clinical, immunophenotypic, and molecular study. *Cancer* **2003**, *98*, 2282-2290, doi:10.1002/cncr.11780.
54. Das, J.K.; Gangopadhyay, A.K. Mycosis fungoides with unusual vitiligo-like presentation. *Indian J. Dermatol, Venereol. Leprol.* **2004**, *70*, 304-306.
55. Fink-Puches, R.; Chott, A.; Ardigo, M.; Simonitsch, I.; Ferrara, G.; Kerl, H.; Cerroni, L. nThe spectrum of cutaneous lymphomas in patients less than 20 years of age. *Pediatr. Dermatol.* **2004**, *21*, 525-533, doi:10.1111/j.0736-8046.2004.21500.x.
56. Gulekon, A.; Ozsoy, E.; Gurer, M.A.; Ataoglu, O. Hypopigmented mycosis fungoides in an adolescent Turkish boy. *Int. J. Dermatol.* **2005**, *44*, 795-796, doi:10.1111/j.1365-4632.2005.02422.x.
57. Hodak, E.; Klein, T.; Gabay, B.; Ben-Amitai, D.; Bergman, R.; Gdalevich, M.; Feinmesser, M.; Maron, L.; David, M. Familial mycosis fungoides: report of 6 kindreds and a study of the HLA system. *J. Am. Acad. Dermatol.* **2005**, *52*, 393-402, doi:10.1016/j.jaad.2003.12.052.
58. Roupe, G. Hypopigmented mycosis fungoides in a child successfully treated with UVA1-light. *Pediatr Dermatol.* **2005**, *22*, 82, doi:10.1111/j.1525-1470.2005.22119.x.
59. Wain, E.M.; Orchard, G.E.; Mayou, S.; Atherton, D.J.; Misch, K.J.; Russell-Jones, R. Mycosis fungoides with a CD56+ immunophenotype. *J. Am. Acad. Dermatol.* **2005**, *53*, 158-163, doi:10.1016/j.jaad.2005.01.133.
60. Hodak, E.; David, M.; Maron, L.; Aviram, A.; Kaganovsky, E.; Feinmesser, M. CD4/CD8 double-negative epidermotropic cutaneous T-cell lymphoma: an immunohistochemical variant of mycosis fungoides. *J. Am. Acad. Dermatol.* **2006**, *55*, 276-284, doi:10.1016/j.jaad.2006.01.020.
61. Hsiao, P.F.; Hsiao, C.H.; Tsai, T.F.; Jee, S.H. Minimal residual disease in hypopigmented mycosis fungoides. *J. Am. Acad. Dermatol.* **2006**, *54*, S198-201, doi:10.1016/j.jaad.2005.08.044.
62. Manzur, A.; Zaidi, S.T. Hypopigmented mycosis fungoides in a 10-year-old boy. *Dermatol. Online J.* **2006**, *12*, 21.
63. Tan, E.S.; Tang, M.B.; Tan, S.H. Retrospective 5-year review of 131 patients with mycosis fungoides and Sezary syndrome seen at the National Skin Centre, Singapore. *Australas. J. Dermatol.* **2006**, *47*, 248-252, doi:10.1111/j.1440-0960.2006.00290.x.
64. Rodriguez, G.; Gonzalez, R.; Gonzalez, D.; Granados, C.; Pinto, R.; Herrera, H.; Gutierrez, L.F.; Hernandez, E.; Lopez, F.; Gomez, Y. [Active search for leprosy and other skin diseases in school children from Agua de Dios, Colombia]. *Rev. Salud Publ. (Bogota, Colombia)* **2007**, *9*, 430-438, doi:10.1590/s0124-00642007000300011.
65. Chuang, G.S.; Wasserman, D.I.; Byers, H.R.; Demierre, M.F. Hypopigmented T-cell dyscrasia evolving to hypopigmented mycosis fungoides during etanercept therapy. *J. Am. Acad. Dermatol.* **2008**, *59*, S121-122, doi:10.1016/j.jaad.2008.06.042.
66. Ozcan, D.; Seckin, D.; Ozdemir, B.H. Hypopigmented macules in an adult male patient. *Clin. Exp. Dermatol.* **2008**, *33*, 667-668, doi:10.1111/j.1365-2230.2008.02834.x.
67. Kim, S.T.; Sim, H.J.; Jeon, Y.S.; Lee, J.W.; Roh, H.J.; Choi, S.Y.; Kim, Y.J.; Suh, K.S. Clinicopathological features and T-cell receptor gene rearrangement findings of mycosis fungoides in patients younger than age 20 years. *J. Dermatol.* **2009**, *36*, 392-402, doi:10.1111/j.1346-8138.2009.00665.x.

68. Ngo, J.T.; Trotter, M.J.; Haber, R.M. Juvenile-onset hypopigmented mycosis fungoides mimicking vitiligo. *J. Cutan. Med. Surg.* **2009**, *13*, 230-233, doi:10.2310/7750.2008.08050.
69. Alsaleh, Q.A.; Nanda, A.; Al-Ajmi, H.; Al-Sabah, H.; Elkashlan, M.; Al-Shemmari, S.; Demierre, M.F. Clinicoepidemiological features of mycosis fungoides in Kuwait, 1991-2006. *Int J. Dermatol.* **2010**, *49*, 1393-1398, doi:10.1111/j.1365-4632.2010.04567.x.
70. Cho-Vega, J.H.; Tschen, J.A.; Duvic, M.; Vega, F. Early-stage mycosis fungoides variants: case-based review. *Ann. Diagn. Pathol.* **2010**, *14*, 369-385, doi:10.1016/j.anndiagpath.2010.06.003.
71. Costa, I.M.; Queiroz Zancanaro, P.C. Hypopigmented patches in a caucasian male: a quiz. *Acta Dermato-venereol.* **2010**, *90*, 109-111, doi:10.2340/00015555-0736.
72. Lawrence, H.; Shao, L.; Horii, K.A. Hypopigmented macules and patches on the extremities of a teenager. *Pediatr. Dermatol.* **2010**, *27*, 197-198, doi:10.1111/j.1525-1470.2010.01118.x.
73. Nanda, A.; AlSaleh, Q.A.; Al-Ajmi, H.; Al-Sabah, H.; Elkashlan, M.; Al-Shemmari, S.; Demierre, M.F. Mycosis fungoides in Arab children and adolescents: a report of 36 patients from Kuwait. *Pediatr. Dermatol.* **2010**, *27*, 607-613, doi:10.1111/j.1525-1470.2010.01129.x.
74. Grover, S.; Verma, R.; Mani, N.S.; Grewal, R.S.; Singh, G.K. Primary Cutaneous T-cell Lymphoma: Two Rare Presentations. *Med. J. Armed Forces India* **2010**, *66*, 73-75, doi:10.1016/S0377-1237(10)80103-0.
75. Khopkar, U.; Doshi, B.R.; Dongre, A.M.; Gujral, S. A study of clinicopathologic profile of 15 cases of hypopigmented mycosis fungoides. *Indian J Dermatol.Venereol Leprol.* **2011**, *77*, 167-173, doi:10.4103/0378-6323.77456.
76. Yazganoglu, K.D.; Topkarci, Z.; Buyukbabani, N.; Baykal, C. Childhood mycosis fungoides: a report of 20 cases from Turkey. *J. Eur. Acad. Dermatol. Venereol.* **2013**, *27*, 295-300, doi:10.1111/j.1468-3083.2011.04383.x.
77. Rueda, X.; Cortés, C.; Acosta, Á. Experiencia de linfomas cutáneos entre enero de 1995 y abril de 2008 en el Instituto Nacional de Cancerología. *Rev. Colomb. Cancerol.* **2011**, *15*, 178-189, doi:10.1016/S0123-9015(12)70048-3.
78. Koorse, S.; Tirumalae, R.; Yeliur, I.K.; Jayaseelan, E. Clinicopathologic profile of hypopigmented mycosis fungoides in India. *Am. J. Dermatopathol.* **2012**, *34*, 161-164, doi:10.1097/DAD.0b013e31822e6877.
79. Rizzo, F.A.; Vilar, E.G.; Pantaleao, L.; Fonseca, E.C.; Magrin, P.F.; Henrique-Xavier, M.; Rochael, M.C. [Mycosis fungoides in children and adolescents: a report of six cases with predominantly hypopigmentation, along with a literature review]. *Dermatol. Online J.* **2012**, *18*, 5.
80. Uhlenhake, E.E.; Mehregan, D.M. Annular hypopigmented mycosis fungoides: a novel ringed variant. *J. Cutan. Pathol.* **2012**, *39*, 535-539, doi:10.1111/j.1600-0560.2012.01880.x.
81. Castano, E.; Glick, S.; Wolgast, L.; Naeem, R.; Sunkara, J.; Elston, D.; Jacobson, M. Hypopigmented mycosis fungoides in childhood and adolescence: a long-term retrospective study. *J. Cutan. Pathol.* **2013**, *40*, 924-934, doi:10.1111/cup.12217.
82. Juhas, E.; English, J.C., 3rd. Hypopigmented patches on the skin. *JAMA* **2013**, *309*, 392-393, doi:10.1001/jama.2012.211717.
83. Seif El Nasr, H.; Shaker, O.G.; Fawzi, M.M.; El-Hanafi, G. Basic fibroblast growth factor and tumour necrosis factor alpha in vitiligo and other hypopigmented disorders: suggestive possible therapeutic targets. *J. Eur. Acad. Dermatol. Venereol.* **2013**, *27*, 103-108, doi:10.1111/j.1468-3083.2011.04368.x.
84. Zhang, J.A.; Yu, J.B. Hypopigmented mycosis fungoides in a chinese woman. *Indian J. Dermatol.* **2013**, *58*, 161, doi:10.4103/0019-5154.108093.
85. Furlan, F.C.; de Paula Pereira, B.A.; da Silva, L.F.; Sanches, J.A. Loss of melanocytes in hypopigmented mycosis fungoides: a study of 18 patients. *J. Cutan. Pathol.* **2014**, *41*, 101-107, doi:10.1111/cup.12262.
86. Furlan, F.C.; Pereira, B.A.; Sotto, M.N.; Sanches, J.A. Hypopigmented mycosis fungoides versus mycosis fungoides with concomitant hypopigmented lesions: same disease or different variants of mycosis fungoides? *Dermatol.* **2014**, *229*, 271-274, doi:10.1159/000363319.
87. Heng, Y.K.; Koh, M.J.; Giam, Y.C.; Tang, M.B.; Chong, W.S.; Tan, S.H. Pediatric mycosis fungoides in Singapore: a series of 46 children. *Pediatr. Dermatol.* **2014**, *31*, 477-482, doi:10.1111/pde.12352.
88. Jimenez Gallo, D.; Albarran Planelles, C.; Linares Barrios, M.; Fernandez Anguita, M.J.; Marquez Enriquez, J.; Rodriguez Mateos, M.E. Treatment of pruritus in early-stage hypopigmented mycosis fungoides with aprepitant. *Dermatol. Ther.* **2014**, *27*, 178-182, doi:10.1111/dth.12113.
89. Fatemi Naeini, F.; Abtahi-Naeini, B.; Sadeghiyan, H.; Nilforoushzadeh, M.A.; Najafian, J.; Pourazizi, M. Mycosis fungoides in Iranian population: an epidemiological and clinicopathological study. *J. Skin Cancer* **2015**, *2015*, 306543, doi:10.1155/2015/306543.

90. Abdel-Halim, M.; El-Nabarawy, E.; El Nemr, R.; Hassan, A.M. Frequency of hypopigmented mycosis fungoides in Egyptian patients presenting with hypopigmented lesions of the trunk. *Am. J. Dermatopathol.* **2015**, *37*, 834-840, doi:10.1097/dad.0000000000000379.
91. Naeini, F.F.; Soghrati, M.; Abtahi-Naeini, B.; Najafian, J.; Rajabi, P. Co-existence of various clinical and histopathological features of mycosis fungoides in a young female. *Indian J. Dermatol.* **2015**, *60*, 214, doi:10.4103/0019-5154.152588.
92. Amin, S.M.; Tan, T.; Guitart, J.; Colavincenzo, M.; Gerami, P.; Yazdan, P. CD8+ mycosis fungoides clinically masquerading as alopecia areata. *J. Cutan. Pathol.* **2016**, *43*, 1179-1182, doi:10.1111/cup.12805.
93. Ichimura, Y.; Sugaya, M.; Morimura, S.; Suga, H.; Sato, S.; Mori, S.; Takahashi, H.; Akutsu, Y. Two cases of CD8-positive hypopigmented mycosis fungoides without TOX expression. *Int. J. Dermatol.* **2016**, *55*, e164-167, doi:10.1111/ijd.13080.
94. Khader, A.; Manakkad, S.P.; Shaan, M.; Pillai, S.S.; Riyaz, N.; Manikoth, P.B.; Kunnummel, M.; Balakrishnan, S. A Clinicopathological Analysis of Primary Cutaneous Lymphomas: A 6-year Observational Study at a Tertiary Care Center of South India. *Indian J. Dermatol.* **2016**, *61*, 608-617, doi:10.4103/0019-5154.193665.
95. Nasu-Tababuchi, M.; Fujimura, T.; Kakizaki, A.; Shido, K.; Hatchome, N.; Kusakari, Y.; Aiba, S. Hypopigmented mycosis fungoides: An immunological investigation of tumor-infiltrating T cells. *Dermatol. Sinica* **2016**, *34*, 96-98, doi:https://doi.org/10.1016/j.dsi.2015.08.006.
96. Paton, D.J.; Van Vliet, C.; Prasad Kumarasinghe, S.; Chan, J.J.; Wood, B.A. Epidermotropic CD8 positive lymphoproliferative diseases: histological and immunophenotypic similarities but markedly differing clinical behaviour. *Pathology* **2016**, *48*, 733-736, doi:10.1016/j.pathol.2016.08.014.
97. Patraquim, C.; Gomes, M.M.; Garcez, C.; Leite, F.; Oliva, T.; Santos, A.; Pinto, A. Childhood Hypopigmented Mycosis Fungoides: A Rare Diagnosis. *Case Rep. Pediatr.* **2016**, *2016*, 8564389, doi:10.1155/2016/8564389.
98. Rowe, B.; Shevchenko, A.; Yosipovitch, G. Leser-Trelat Sign in Tumor-Stage Mycosis Fungoides. *Dermatol. Online J.* **2016**, *22*.
99. Bisherwal, K.; Singal, A.; Pandhi, D.; Sharma, S. Hypopigmented Mycosis Fungoides: Clinical, Histological, and Immunohistochemical Remission Induced by Narrow-band Ultraviolet B. *Indian J. Dermatol.-* **2017**, *62*, 203-206, doi:10.4103/ijd.IJD_365_16.
100. Cervini, A.B.; Torres-Huamani, A.N.; Sanchez-La-Rosa, C.; Galluzzo, L.; Solernou, V.; Digiorge, J.; Rubio, P. Mycosis Fungoides: Experience in a Pediatric Hospital. *Actas Dermosifiliogr.* **2017**, *108*, 564-570, doi:10.1016/j.ad.2017.01.008.
101. Pradhan, D.; Jedrych, J.J.; Ho, J.; Akilov, O.E. Hypopigmented Mycosis Fungoides with Large Cell Transformation in a Child. *Pediatr. Dermatol.* **2017**, *34*, e260-e264, doi:10.1111/pde.13233.
102. Rodney, I.J.; Kindred, C.; Angra, K.; Qutub, O.N.; Villanueva, A.R.; Halder, R.M. Hypopigmented mycosis fungoides: a retrospective clinicohistopathologic study. *J. Eur. Acad. Dermatol. Venereol.* **2017**, *31*, 808-814, doi:10.1111/jdv.13843.
103. Virmani, P.; Levin, L.; Myskowski, P.L.; Flores, E.; Marchetti, M.A.; Lucas, A.S.; Pulitzer, M.; Horwitz, S.; Trippett, T.; Moskowitz, A., et al. Clinical Outcome and Prognosis of Young Patients with Mycosis Fungoides. *Pediatr. Dermatol.* **2017**, *34*, 547-553, doi:10.1111/pde.13226.
104. Binamer, Y. Cutaneous T-cell lymphoma in Saudi Arabia: retrospective single-center review. *Ann. Saudi Med.* **2017**, *37*, 212-215, doi:10.5144/0256-4947.2017.212.
105. Martinez-Escala, M.E.; Kantor, R.W.; Cices, A.; Zhou, X.A.; Kaplan, J.B.; Pro, B.; Choi, J.; Guitart, J. CD8(+) mycosis fungoides: A low-grade lymphoproliferative disorder. *J. Am. Acad. Dermatol.* **2017**, *77*, 489-496, doi:10.1016/j.jaad.2017.05.015.
106. Abdolkarimi, B.; Sepaskhah, M.; Mokhtari, M.; Aslani, F.S.; Karimi, M. Hypo-pigmented mycosis fungoides is a rare malignancy in pediatrics. *Dermatol. Online J.* **2018**, *24*.
107. Amorim, G.M.; Niemeyer-Corbellini, J.P.; Quintella, D.C.; Cuzzi, T.; Ramos, E.S.M. Hypopigmented mycosis fungoides: a 20-case retrospective series. *Int. J. Dermatol.* **2018**, *57*, 306-312, doi:10.1111/ijd.13855.
108. Ferreira, B.R.; Ramos, L.; Cardoso, J.C.; Reis, J.P.; Tellechea, O. Hypopigmented patches in childhood: do not forget mycosis fungoides. *Clin. Exp. Dermatol.* **2019**, *44*, 588-589, doi:10.1111/ced.13807.
109. Stierman, S.; Bedford-Lyon, N. Hypopigmented discoloration on the thigh. *Cutis* **2018**, *101*, E4-E6.

110. Joseph, M.X.; Brown, A.D.; Davis, L.S. The importance of lymph node examination: Simultaneous diagnosis of hypopigmented mycosis fungoides and follicular B-cell lymphoma. *JAAD Case Rep.* **2018**, *4*, 590-592, doi:10.1016/j.jdcr.2018.05.017.
111. Vilas Boas, P.; Hernandez-Aragues, I.; Suarez-Fernandez, R.; Campos-Dominguez, M. Hypopigmented patches on the buttocks of a 7-year-old boy. *Clin. Exp. Dermatol.* **2018**, *43*, 485-487, doi:10.1111/ced.13385.
112. Yang, M.Y.; Jin, H.; You, H.S.; Shim, W.H.; Kim, J.M.; Kim, G.W.; Kim, H.S.; Ko, H.C.; Kim, B.S.; Kim, M.B. Hypopigmented Mycosis Fungoides Treated with 308 nm Excimer Laser. *Ann. Dermatol.* **2018**, *30*, 93-95, doi:10.5021/ad.2018.30.1.93.
113. Youssef, R.; Mahgoub, D.; Zeid, O.A.; Abdel-Halim, D.M.; El-Hawary, M.; Hussein, M.F.; Morcos, M.A.; Aboelfadl, D.M.; Abdelkader, H.A.; Abdel-Galeil, Y., et al. Hypopigmented Interface T-Cell Dyscrasia and Hypopigmented Mycosis Fungoides: A Comparative Study. *Am. J. Dermatopathol.* **2018**, *40*, 727-735, doi:10.1097/DAD.0000000000001187.
114. Park, A.Y.; Sim, C.Y.; Lee, S.Y.; Lee, J.S.; Hong, S.A.; Kim, J.E. A case of hypopigmented mycosis fungoides successfully treated with 311 nm narrowband ultraviolet B phototherapy. *Dermatol. Sinica* **2018**, *36*, 207-210, doi:https://doi.org/10.1016/j.dsi.2018.04.004.
115. Ito, A.; Sugita, K.; Ikeda, A.; Yamamoto, O. CD4/CD8 Double-negative Mycosis Fungoides: A Case Report and Literature Review. *Yonago Acta Med.* **2019**, *62*, 153-158, doi:10.33160/yam.2019.03.021.
116. Jaque, A.; Mereniuk, A.; Walsh, S.; Shear, N.H.; Sade, S.; Zagorski, B.; Alhusayen, R. Influence of the phenotype on mycosis fungoides prognosis, a retrospective cohort study of 160 patients. *Int. J. Dermatol.* **2019**, *58*, 933-939, doi:10.1111/ijd.14391.
117. Lim, H.L.J.; Tan, E.S.T.; Tee, S.I.; Ho, Z.Y.; Boey, J.J.J.; Tan, W.P.; Tang, M.B.Y.; Shen, L.; Chan, Y.H.; Tan, S.H. Epidemiology and prognostic factors for mycosis fungoides and Sezary syndrome in a multi-ethnic Asian cohort: a 12-year review. *J. Eur. Acad. Dermatol. Venereol.* **2019**, *33*, 1513-1521, doi:10.1111/jdv.15526.
118. Chen, J.; Yu, H.; Yao, Z. Coexistence of hypopigmented mycosis fungoides and erythema dyschromicum perstans in a 3-year-old Chinese girl. *J. Eur. Acad. Dermatol. Venereol.* **2019**, *33*, e492-e494, doi:10.1111/jdv.15833.
119. Geller, S.; Lebowitz, E.; Pulitzer, M.P.; Horwitz, S.M.; Moskowitz, A.J.; Dusza, S.; Myskowski, P.L. Outcomes and prognostic factors in African American and black patients with mycosis fungoides/Sezary syndrome: Retrospective analysis of 157 patients from a referral cancer center. *J. Am. Acad. Dermatol.* **2019**, 10.1016/j.jaad.2019.08.073, doi:10.1016/j.jaad.2019.08.073.
120. Kalay Yildizhan, I.; Sanli, H.; Akay, B.N.; Surgun, E.; Heper, A. CD8(+) cytotoxic mycosis fungoides: a retrospective analysis of clinical features and follow-up results of 29 patients. *Int. J. Dermatol.* **2020**, *59*, 127-133, doi:10.1111/ijd.14689.

© 2020 by the authors. Licensee MDPI, Basel, Switzerland. This article is an open access article distributed under the terms and conditions of the Creative Commons Attribution (CC BY) license (http://creativecommons.org/licenses/by/4.0/).
